# Misrepresentation of group contributions undermines conditional cooperation in a human decision making experiment

**DOI:** 10.1038/s41598-022-16613-5

**Published:** 2022-07-19

**Authors:** Pieter van den Berg, Siyuan Liu, Tom Wenseleers, Jianlei Zhang

**Affiliations:** 1 Department of Biology, Evolutionary Modelling Group, KU Leuven, B-3000 Leuven, Belgium; 2 Department of Microbial and Molecular Systems, Evolutionary Modelling Group, KU Leuven, B-3000 Leuven, Belgium; 3 Department of Biology, Lab of Socioecology and Social Evolution, KU Leuven, B-3000 Leuven, Belgium; 4 Department of Automation, College of Artifificial Intelligence, Nankai University, Tianjin, 300071 China; 5 Tianjin Key Laboratory of Intelligent Robotics, Nankai University, Tianjin, 300071 China

**Keywords:** Social evolution, Human behaviour

## Abstract

Cooperative behaviour can evolve through conditional strategies that direct cooperation towards interaction partners who have themselves been cooperative in the past. Such strategies are common in human cooperation, but they can be vulnerable to manipulation: individuals may try to exaggerate their past cooperation to elicit reciprocal contributions or improve their reputation for future gains. Little is known about the prevalence and the ramifications of misrepresentation in human cooperation, neither in general nor about its cultural facets (self-sacrifice for the group is valued differently across cultures). Here, we present a large-scale interactive decision making experiment (N = 870), performed in China and the USA, in which individuals had repeated cooperative interactions in groups. Our results show that (1) most individuals from both cultures overstate their contributions to the group if given the opportunity, (2) misrepresentation of cooperation is detrimental to cooperation in future interactions, and (3) the possibility to build up a personal reputation amplifies the effects of misrepresentation on cooperation in China, but not in the USA. Our results suggest that misrepresentation of cooperation is likely to be an important factor in (the evolution of) human social behaviour, with, depending on culture, diverging impacts on cooperation outcomes.

## Introduction

In social dilemma situations, individuals have to choose between actions that are in their own short-term interest and actions that are in the group interest. Even though acting selfishly is most profitable in the short term, it has been established that evolution can promote behaviours that favour the group interest^1,2^. For example, evolution can lead to high cooperation rates through the emergence of conditional cooperation, where individuals reciprocate the cooperative acts of others^3–8^. Evolution can also promote cooperation through reputation mechanisms, where individuals have an incentive to act cooperatively because it enhances their reputation, which in turn affords opportunities for social or material benefits in the future^6,7,9–12^. Such reputation mechanisms can even work if individuals interact in temporary groups and have limited access to the previous cooperation of individuals in other groups^13^. Experimental studies have shown that both are relevant for human behaviour: individuals are likely to reciprocate cooperative acts, and also to direct altruism towards those whom they have seen acting cooperatively towards others^14–20^.

Both reciprocal cooperation and reputation-based cooperation rely on the perception of others: an unseen cooperative act will not confer future benefits, since it cannot be reciprocated or enhance one’s reputation. This provides room for manipulation: if possible, it is most profitable to *act* selfishly (to reap short-term gains) while *appearing* to act in the common interest (to reap medium- and long-term gains, through reciprocal cooperation of interaction partners and reputation benefits). Several theoretical studies have predicted that there is scope for the evolution of strategies that attempt to deceive interaction partners about past actions or intentions^21–23^, and that such strategies can undermine cooperation^24^. It has indeed been shown that people are motivated to portray themselves as more cooperative than they are intrinsically motivated to act. Already at a young age, children act more generously and are less likely to break rules for selfish purposes when they know they are being watched^25,26^. It has been shown across a range of experimental paradigms that people are less likely to act selfishly (and less likely to lie about acting selfishly) if others are watching^27–30^.

**Figure 1:**
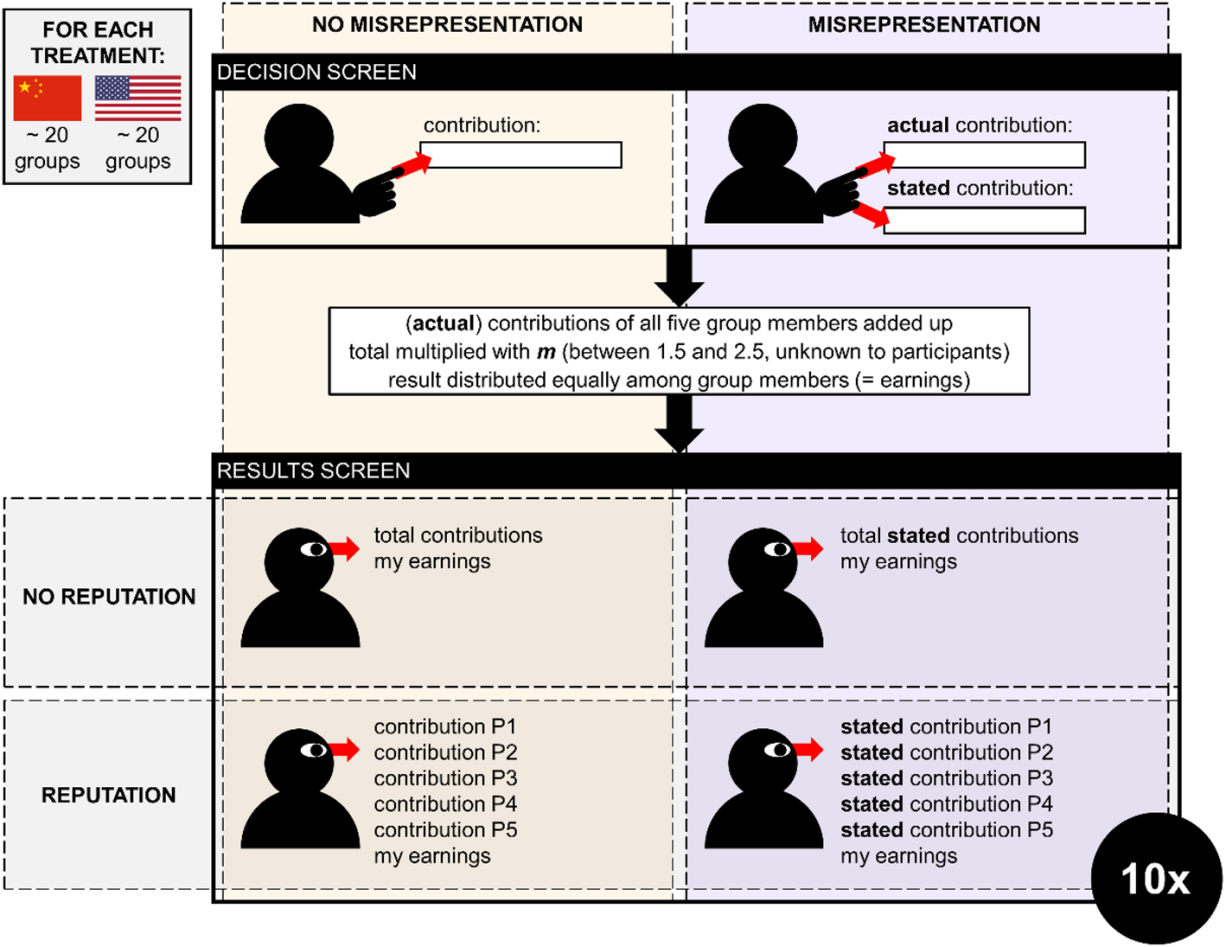
Overview of the experimental design. The experiment was run online and consisted of four experimental treatments: all combinations of the possibility for misrepresentation (with or without) and the possibility for reputation (with or without). Participants received an endowment of five Points and had to enter a contribution to the group project on the ‘Decision screen’. They entered either their actual contribution (treatments without misrepresentation, left side of figure in yellow) or both an actual contribution and a stated contribution (treatments with misrepresentation, right side of the figure in purple). On the ‘Results screen’, participants were shown the actual contributions (treatments without misrepresentation) or the stated contributions (treatments with misrepresentation) of the group members. In the treatments without reputation, individuals were only shown the total (stated) contributions of all group members, whereas they were shown all individual (stated) contributions in the treatments with reputation. In all treatments, the Results screen also showed the participant’s own earnings of the round, which consisted of an equal share of the total Points invested in the group project multiplied with *m* (which could take any value between 1.5 and 2.5, see “Methods”) and the Points the individual did not invest in the group project. After the Results screen, the participants started a new round on the Decision screen (in the same group), for a total of ten rounds.

In summary, humans have incentives to overstate the contributions that they have made in the group interest, and they also have both the motivations and the capability to do so. However, little is known about the extent to which humans actually engage in such misrepresentation and its consequences for continued social interactions. Here, we present a large-scale online interactive decision making experiment (pre-registered at https://osf.io/c2674) to investigate this. We do this by having participants interact repeatedly in public goods games (PGGs) and, depending on the experimental treatment, allowing them to misrepresent their contributions to the public good (See Fig. 1 and “Methods” for an overview of the experimental setup). This allows us to obtain an overview of the degree to which participants misrepresent, and to investigate how the possibility of misrepresentation affects the cooperation levels throughout the progression of the interaction. Our expectation was that the possibility of misrepresentation undermines cooperation, because it leads individuals to distrust their interaction partners’ contribution statements, thereby harming their willingness to reciprocate.

Misrepresentation of cooperation can both be motivated by the prospect of eliciting future cooperation from interaction partners and by the desire to enhance one’s own reputation. There are reasons to suspect that especially the latter motivation may be subject to cultural differences. Culturally derived norms affect cooperative behaviour in various contexts^31–38^ and cultures differ in the degree to which they value self-sacrifice for the common good^39,40^. This means that the cultural environment likely impacts the potential reputation benefits that can be gained by overstating contributions to a public good. Specifically, individuals in collectivist cultures, where self-sacrifice is highly valued, may have more reputation benefits to gain by (mis)presenting themselves as cooperative than people in individualist cultures, but they may also arouse more suspicion by attempting to do so.

In addition to investigating the overall incidence and impact on cooperation of misrepresentation, this study also assesses the relative importance of reputation concerns in the context of misrepresentation between an individualist culture (USA) and a collectivist culture (China). Specifically, we expected reputation concerns to play a stronger role in China than in the USA, more strongly enhancing cooperation in the absence of misrepresentation but also more strongly undermining cooperation its presence.

## Results

### Prevalence of misrepresentation

**Figure 2:**
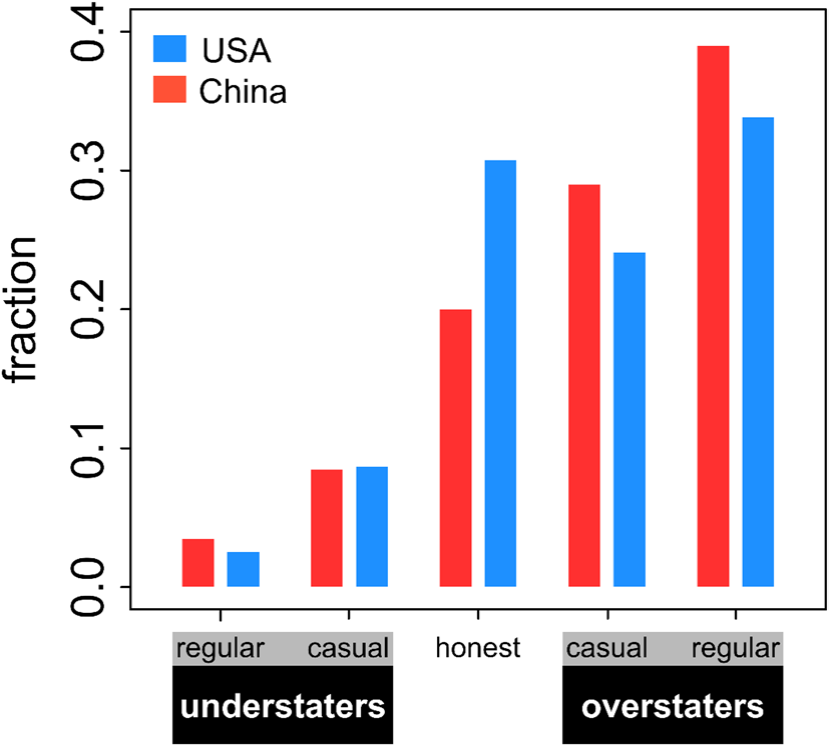
Most participants across both cultures tend to overstate the contributions they made to the group project. Bars show the fractions of individuals that were ‘understaters’ (understating their contribution more often than they overstated them) ‘honest’ (individuals who understated and overstated equally often, which are mostly made up of individuals who never misrepresented at all) and ‘overstaters’ (overstating more often than understating). ‘Regular’ understaters are defined as individuals who understated in at least five more rounds than they overstated, while ‘casual’ understaters are individuals who understated in fewer than five more rounds than they overstated (the same reasoning applies to overstaters). The figure shows pooled data for the treatments with and without the possibility for reputation.

Figure 2 shows that many individuals in both China and the USA made use of the opportunity to misrepresent their contributions in the treatments where this was possible. Chinese subjects misrepresented their group project contributions in a somewhat larger fraction of instances (57.3%) than American subjects (51.0%), but this difference was not significant (mixed binomial glm: P = 0.15). If they misrepresented, Chinese and American subjects overstated to a similar degree on average (China: 0.34, USA: 0.35), but American participants were more likely to state that they contributed their entire endowment when they actually contributed nothing (10.9% of all cases for American subjects, 4.3% of all cases for Chinese subjects; mixed binomial glm: P = 0.002). Figure 2 shows that there were clear individual differences in misrepresentation across both cultures: sizable fractions never mispresented or overstated their contributions as often as they understated (20% in China, 31% in the USA), relatively small but considerable fractions tended to understate their contributions (12% in China, 11% in the USA), and a majority of individuals in both cultures tended to overstate their contributions (of which many overstated in the majority of the interaction rounds).

### Consequences of misrepresentation

**Figure 3:**
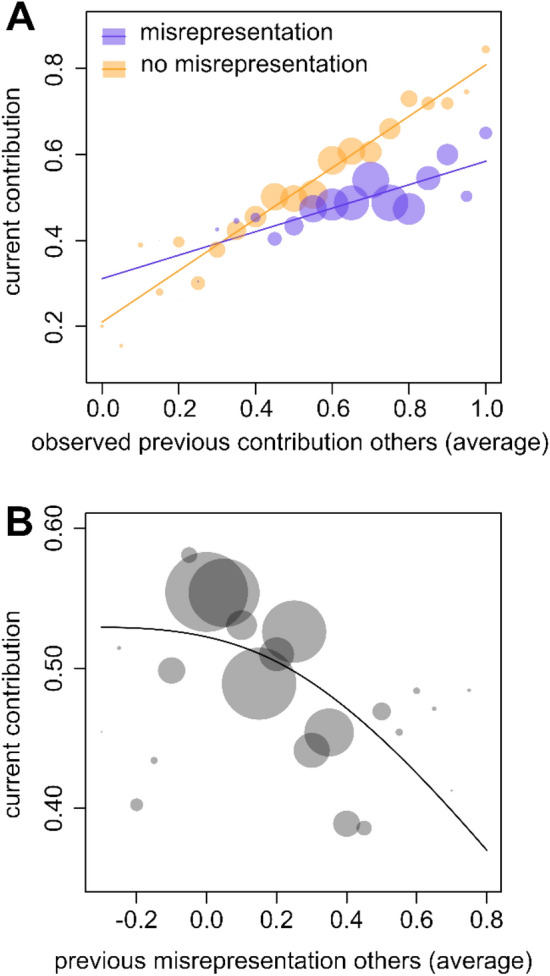
Misrepresentation undermines cooperation by weakening conditional cooperation and eliciting lower contributions. (**A**) If misrepresentation was possible, individuals were less responsive to the contributions of the other group members than in the absence of misrepresentation opportunities. Circles indicate how much individuals contributed on average depending on the average observed previous contribution of their fellow group members (i.e. stated contributions in case of the misrepresentation treatments). The radius of the circles indicate number of observations. The lines are given by a simple linear regression that included only misrepresentation, the previous average contribution of the fellow group members, and their interaction as predictors. (**B**) Increasing misrepresentation of fellow group members in the previous round led to decreasing contributions. The circles indicate the average contribution made depending on the average degree of misrepresentation of the other group members in the previous round. The radius of the circles indicate number of observations. The line is given by a simple linear regression that included only a natural cubic spline with two degrees of freedom in function of the previous average misrepresentation of the fellow group members as a predictor (this is consistent with how this variable was modelled in our overall statistical analysis, see “Methods”). Negative values indicate cases where individuals underrepresented their contributions, whereas positive values indicate overrepresentations.

Misrepresentation had appreciable effects on cooperation rates in our experiment. Participants tended to be conditionally cooperative (i.e. they tended to contribute more if their interaction partners contributed more in the previous round), but this conditionality was significantly weaker if misrepresentation was possible (see Fig. 3A; linear mixed model [Model 1; see “Methods”], significant interaction effect between misrepresentation treatment and the observed contribution of the interaction partners in the previous round: P < 0.001). In the absence of misrepresentation, participants responded to every 1-point increase in contributions by their fellow group members with an average 0.60-point increase in their own contribution in the next round. When misrepresentation was possible, they only responded with a 0.27-point increase. This effect was significant when considering the data from both cultures separately (linear mixed models, China: P < 0.001; USA: P = 0.002).

In addition, even though misrepresentation was not directly observable, individuals tended to contribute less if their interaction partners misrepresented more in the previous round (see Fig. 3B; linear mixed model [Model 2]: P < 0.001; this effect showed similar patterns for subsets of both cultures, but was not significant in China: P = 0.208; USA: P = 0.008). This may reflect a mistrust of statements of high contributions when individuals are aware that these statements may be misrepresentations.

Finally, based on Model 3, we concluded that the degree to which individuals misrepresented was not impacted by the degree of misrepresentation of fellow group members in the previous round (see Supplementary Information for all estimates and effect plots of this model).

### Cultural differences

**Figure 4:**
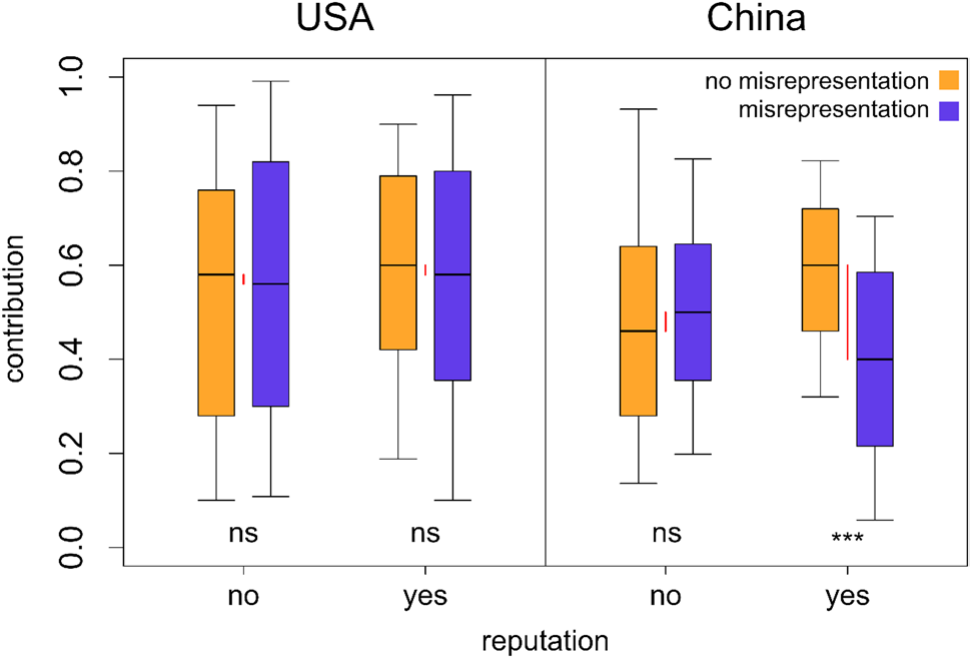
Reputation effects resulted in large contribution differences between the treatments with and without misrepresentation in China, but not in the USA. Boxplots show median, interquartile range (box) and the 10% and 90% quantiles (whiskers). Red lines show the differences in mean contribution rates between the treatments with and without misrepresentation. ‘ns’ (P > 0.05) and ‘***’ (P < 0.001) indicate the significance of Tukey HSD based on a linear mixed model; see “Methods” for details.

Although misrepresentation was both common and consequential for cooperation in China as well as in the USA, we also observed a difference between the behaviour of Chinese and American subjects, depending on whether it was possible to build up an individual reputation or not. The possibility of reputation hardly affected the contributions of the American subjects, whereas it had a clear impact on Chinese subjects (see Fig. 4). Specifically, the possibility to build up a reputation caused Chinese participants to contribute significantly more if they could not misrepresent than if they could (Tukey HSD based on linear mixed model, z-ratio: 4.066, P < 0.001; rightmost pair of bars in Fig. 4). This effect was completely absent if building up a reputation was not possible (z-ratio: − 0.102, P = 1.000; third pair of bars in Fig. 4). In contrast, the possibility for building up a reputation did not affect the contributions of American subjects (P > 0.986 for all comparisons between experimental treatments; two leftmost pairs of bars in Fig. 4). In line with this, we observed a significant two-way interaction between misrepresentation treatment, reputation treatment, and culture (linear mixed model [Model 1], P = 0.041).

## Discussion

Our study clearly showed that many individuals in both China and the USA are highly motivated to misrepresent contributions to a group effort. In addition, the distribution of individual misrepresentation tendencies is remarkably similar in both cultures, suggesting that our results may be reflective of more general human tendencies to misrepresent cooperation efforts. Specifically, 77% of participants across both cultures misrepresented their contributions at least once (81% of Chinese subjects; 73% of American subjects), 60% of participants misrepresented their contributions in at least half of all rounds (65% in of Chinese subjects; 56% of American subjects), and 19% of individuals misrepresented *all* of their contributions (18% of Chinese subjects; 20% of American subjects). This suggests that misrepresentation of contributions to group efforts is likely to be pervasive in social dilemmas where it is possible to credibly do so, and that seeking out opportunities to misrepresent contributions may be an important part of participating in social interactions.

Our results show that there are clear individual differences in misrepresentation in both China and the USA (see Fig. 2). These individual differences with respect to the frequency and degree with which people misrepresent may be associated with personality differences. For example, a recent study suggests that individuals who score high on conscientiousness or low on neuroticism are less likely to tell self-serving lies^41^. From an evolutionary perspective, this individual variation is perhaps not surprising: deception will yield the greatest benefits when rare^42^ since selection for counter-adaptations increases with its frequency. Gaining more insight in the underlying causes of these individual differences in misrepresentation behaviour is an important piece of the puzzle of understanding how tensions between individual and group interest are resolved in social interactions.

We also observed that reputation concerns impacted contributions in China, but not in the USA. If reputations concerns could play a role, Chinese subjects contributed less when misrepresentation was possible then if it was not, whereas we observed no such effect if reputation concerns were impossible. In contrast, reputation concerns had no effect on contributions in the USA, regardless of the possibility for misrepresentation. These results may reflect underlying cultural differences in individualism and collectivism: being from a collectivist culture that puts relatively high value on self-sacrifice for the common good^39,40^, the Chinese participants may have been more eager to establish a cooperative reputation. In the absence of misrepresentation, such a reputation reflects real cooperativeness and would therefore be effective in eliciting future contributions from other players as well. In contrast, when misrepresentation was possible, Chinese participants may have been particularly aware of the possibility that their interaction partners might be eager to establish a positive reputation through deception, leading to a stronger undermining of reciprocal cooperation as a result.

It is well-established that cooperation can evolve through the emergence of conditional strategies that either directly and indirectly (through reputation mechanisms) reciprocate earlier cooperation of interaction partners. Our results suggest that the potential for misrepresentation may be relevant to the evolution of such mechanisms, at least in humans. The potential for misrepresentation may lead to adaptations to misrepresent, and these may in turn select for counter-adaptations to prevent, unveil, or punish misrepresentations, possibly giving rise to complex evolutionary dynamics. The full implications of misrepresentation for the evolution of cooperation are yet unclear, which provides potentially interesting opportunities for further research.

Our study provides some points of departure for enhancing cooperative outcomes in public goods dilemmas. First, based on our finding that the possibility for misrepresentation undermines (conditional) cooperation, cooperation may be augmented by restricting opportunities for mispresenting the contributions that individuals have made (e.g. by maintaining public records of actual contributions). Second, our results show that the possibility for reputation actually further undermined cooperation when misrepresentation was possible in China. Hence, if it is not possible to restrict misrepresentation opportunities, it may be effective to restrict possibilities for broadcasting one’s personal contribution instead. However, this is an intervention that would only be effective in some cultures—based on our study, it could be effective in China but not in the USA. In any case, our study shows that misrepresentation of contributions to the common interest is likely to be an important phenomenon across cultures, with—depending on the specifics of the cultural context—potentially wide-ranging consequences.

## Methods

### Experimental design

Our experiment was a between-subjects design of four experimental treatments in both cultures, in each of which individuals had repeated cooperative interactions in groups. The treatments comprised all four combinations of the possibility of misrepresentation (possible or not possible) and the possibility of establishing an individual reputation (again, possible or not possible). See Fig. 1 for a schematic overview of the experimental design.

Participants (N = 870) played a total of ten rounds of the Public Goods Game (PGG) within the same group of five participants (they were aware that the groups remained fixed). In each round, all group members received an endowment of five Points, and were simultaneously faced with a ‘Decision screen’ in which they had to decide how many Points they would contribute to the group project (the maximum contribution has been rescaled to 1 in this paper). The total contribution to the group project was then multiplied with a multiplication factor *m* and distributed equally to all group members (irrespective of contribution). The value of *m* was variable between rounds, but always between 1.5 and 2.5. Participants were not informed of the exact value of *m* in any given round, but they were informed of its possible range in the instructions before the start of the experiment. This uncertainty in the value of *m* was implemented to prevent participants from deducing the degree of misrepresentation by their fellow group members.

Once all group members had confirmed their contributions in the Decision screen, they were presented with the ‘Results screen’, which summarised the outcome of the PGG (the information on this screen was different between the experimental treatments—see below and Fig. 1). After the Results screen, they were again presented with a Decision screen for the next round. After ten rounds, participants filled out a brief questionnaire eliciting some basic demographic information, and were subsequently informed of their total earnings (they were paid through the recruitment platform). All participants received a ‘flat fee’ of $2.00/RMB 7.50, and an extra payment that was proportional to the number of Points accumulated during the experiment (100 Points = $4/RMB 15).

We ran four experimental treatments both in China and in the USA, aiming for at least 20 groups of 5 individuals per treatment. Our experimental treatments comprised all four combinations of the possibility of misrepresentation (with and without) and the possibility of reputation effects (with and without; see Fig. 1 for a schematic overview of the experimental design). In the treatments without the possibility of misrepresentation, participants simply saw the actual contributions of their fellow group members on the Results screen. In the treatments with the possibility of misrepresentation, participants had to enter both an actual contribution and a stated contribution on the Decision screen, and the stated contributions were shown on the Results screen. In the treatments with the possibility for reputation effects, participants saw all individual (stated) contributions of their fellow group members on the Results screen. In the treatments without the possibility for reputation effects, they only saw the total (stated) group contribution. In addition, the Results screen in all treatments also showed the total Points in the group project after multiplication with the multiplication factor and the number of Points the individual earned in this round (both the Points kept for themselves and the share of the group project; see Supplementary Information for example Results screens of each experimental treatment).

Participants had a maximum of 20 s for each decision and for consulting the Results screen, except in the first two rounds, when they had some more time (35 s) to allow them to get used to the environment. If a participant did not confirm their decision within the time limit, they automatically made a random contribution to the group project and (in the misrepresentation treatments) also made a random stated contribution (such decisions were excluded from analysis). If participants failed to make a decision in time more than twice, they were removed from the experiment (in online interactive experiments, this typically indicates that the participant is no longer actively participating).

In online experiments, dropout inevitably occurs. In our data analysis, we only included groups in which all five participants had made active decisions in at least seven out of ten rounds. This criterion caused us to exclude 63 groups (26.5%) from analysis while retaining at least 20 groups for each experimental treatment, for a total of 174 groups that were included in the analysis. Specifically, our analysis contains 20 groups for all four treatments in China except the treatment without misrepresentation or reputation, for which we had 21 groups, and in the USA we had 24 groups for the treatment with misrepresentation and reputation, 21 groups with misrepresentation and without reputation, 23 groups without misrepresentation and with reputation, and 25 groups without misrepresentation and without reputation.

### Participants

Participants in the USA (N = 465) were recruited through the online labour platform Amazon Mechanical Turk. 60.4% of USA participants were male and their median age was 35 (s.d. = 10.6; min age = 18; max age = 69). Participants in China (N = 405) were recruited through an online labour platform mostly focused on tasks related to training artificial intelligence algorithms with more than 4 million registered users, and the experiments were run on the Alibaba cloud (contact the authors for more details). 36.4% of Chinese participants were male and their median age was 30 (s.d. = 7.2; min age = 18; max age = 55). Experimental sessions lasted around 15–20 min, American participants earned on average $4.82 (s.d. = $0.54; min = $3.18; max = $6.32) and Chinese participants earned on average RMB 17.65 (s.d. = RMB 1.73; min = RMB 11.49, max = RMB 23.75). The earnings in China and the USA were similar when correcting for purchasing power. All procedures were approved by Social and Societal Ethics Committee of KU Leuven (SMEC; file number G-2020-1818-R2(MAR)), all methods were performed in accordance with the relevant guidelines and regulations, and all participants gave informed consent of their participation.

After recruitment on the respective labour platforms, participants clicked a link that led them to our interactive experimental platform (programmed in LIONESS Lab^43^). There, they received detailed instructions, completed a quiz to test their comprehension of the experimental set-up (see Supplementary Information for details), and played a test trial of two rounds to let them get used to the experimental environment without consequences for their earnings. After this, the actual experimental session would begin.

### Statistical analysis

We performed our statistical analysis in accordance with the procedures outlined in the pre-registration of our experiment (https://osf.io/c2674). First, we built a linear mixed model (Model 1) to investigate which factors affected contribution rates in our experiment by initially including the following predictor variables: misrepresentation treatment, reputation treatment, culture (China or USA), the observed contribution of the interaction partners in the previous round (this was the actual contribution in the treatments without misrepresentation and the stated contribution in the treatments with misrepresentation), the round number, all one-way interactions between these variables, and the two-way interaction between misrepresentation treatment, reputation treatment and culture. In addition, we included individual nested in group as a random intercept. We investigated models in which the continuous predictors (round number and observed contribution of the interaction partners in the previous round) were modelled in function of natural cubic splines with varying degrees of freedom, and chose the best model based on BIC. The outcome was that all continuous variables were modelled as linear predictors (i.e., non-spline). We next performed a stepwise backward elimination of variables based on BIC on this model to obtain our final model (with the restriction that the two-way interaction and its constituent parts be retained). The final model retained the main effects of all initial variables, the two-way interaction and its underlying one-way interactions, and one-way interactions between misrepresentation treatment and the observed contribution of the interaction partners in the previous round, between culture and round number, and between culture and the observed contribution of the interaction partners in the previous round (see Supplementary Information for all estimates and effect plots).

Second, we built a linear mixed model (Model 2) to investigate how the degree of misrepresentation affected contributions in our experiment. We used a similar strategy as described above, but now only used the data from the treatments where misrepresentation was possible and hence did not include ‘misrepresentation treatment’ or any of its interaction effects as a predictor in the model. In addition we now included the average misrepresentation of all interaction partners in the previous round as a predictor in the model. Based on a similar procedure as above, the misrepresentation of all other interaction partners in the previous round was modelled in function of a natural cubic spline with two degrees of freedom, and the other continuous predictors were modelled as linear (non-spline) predictors. After backwards elimination, the final model retained the main effects of culture, round number, previous contribution of all interaction partners, and previous misrepresentation of all interaction partners, and the one-way interaction effects between culture and round number, and between culture and the observed contribution of the interaction partners in the previous round.

Third, we built a linear mixed model (Model 3) to investigate how the degree of misrepresentation affected subsequent misrepresentation in our experiment. Again, we used a similar strategy as for Models 1 and 2, only using the data from the misrepresentation treatments. Because we were especially interested in the main effect of the degree of misrepresentation, we removed interactions of this variable with other variables from the final model to assess its main effect. The final model included culture, round number, the previous average misrepresentation of all other group members, the observed previous average contribution of all other group members, and the interaction between culture and the previous average contribution of all other group members. The final model did not include spline-terms.

## Supplementary Information

Supplementary Information.

## Data Availability

All data are available at https://osf.io/pbhft/.
