# Supplementary material for: Misrepresentation of group contributions undermines conditional cooperation in a human decision making experiment
Source: Sci Rep. 2022 Jul 19;12:12320. doi: 10.1038/s41598-022-16613-5 (PMC9296641; doi:10.1038/s41598-022-16613-5)
Supplement: Supplementary file 1 — Supplementary Information. [file 41598_2022_16613_MOESM1_ESM.pdf]

Supplementary Information accompanying the manuscript

Misrepresentation of group contributions undermines conditional cooperation in a human decision making experiment

Pieter van den Berg, Siyuan Liu, Tom Wenseleers, Jianlei Zhang*

*Corresponding author. Email: jianleizhang@nankai.edu.cn

This PDF file includes:

Example screens of experimental software	2
Comprehension questions to test participants	20
Details of statistical models	21
Graphs of contributions over time	37

1. Example screens of experimental software

Supplementary Figures S1 to S18 show example screens of the experimental software. The screens below are all in English, so they are the ones that were used for the part of the experiment that was run in the USA. The Chinese screens were direct translations of these.

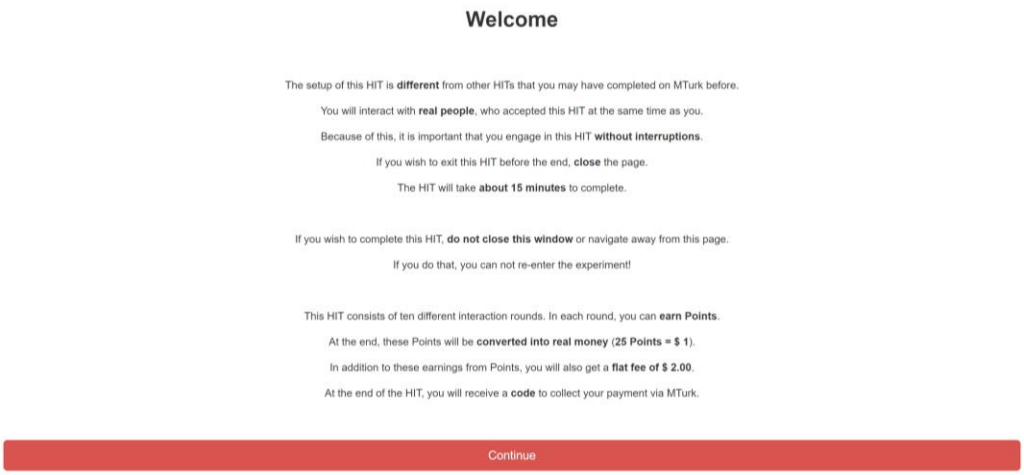

Fig. S1. Welcome screen. This is the first screen the participants saw after entering our experiment from Amazon Mechanical Turk.

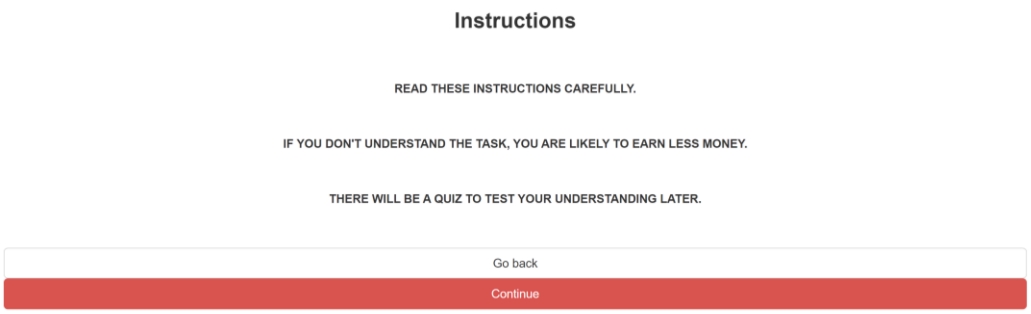

Fig. S2. Instructions screen 1.

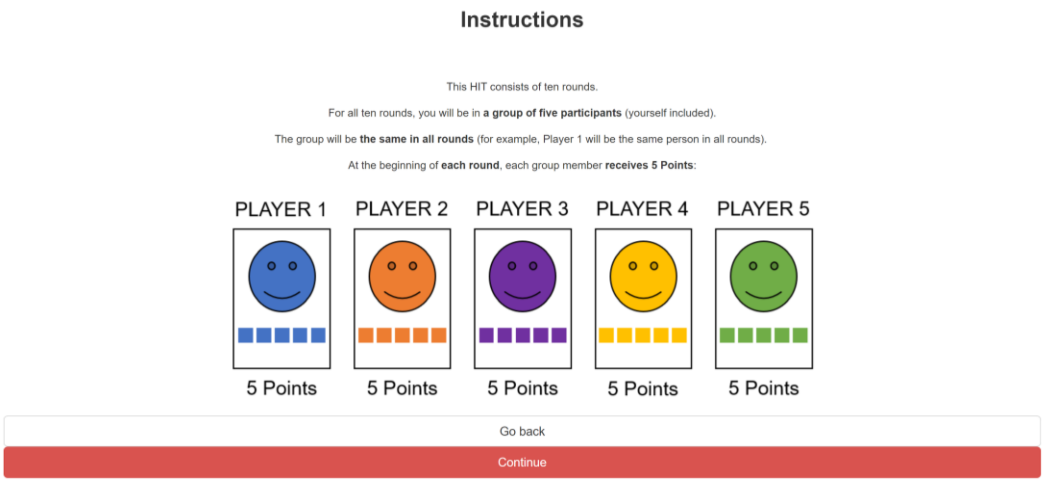

Fig S3. Instructions screen 2.

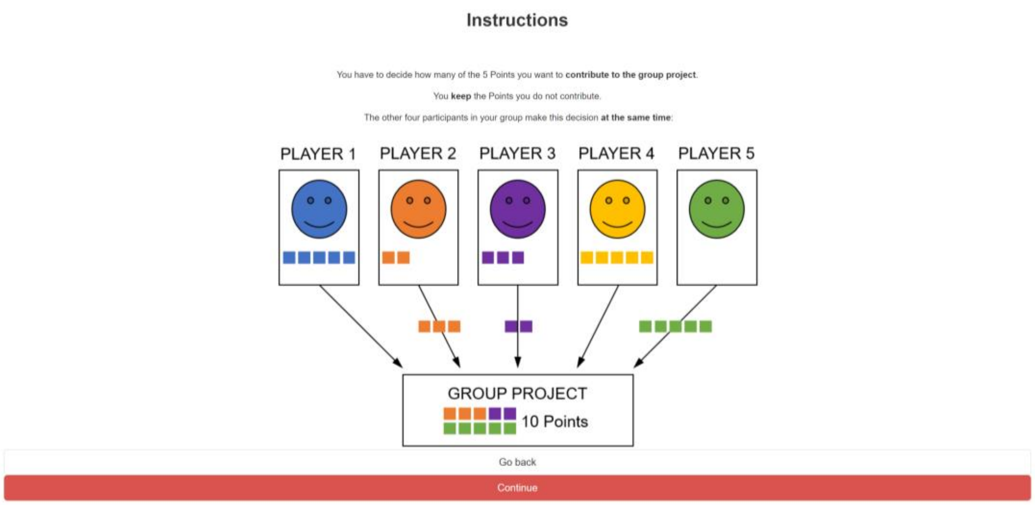

Fig S4. Instructions screen 3.

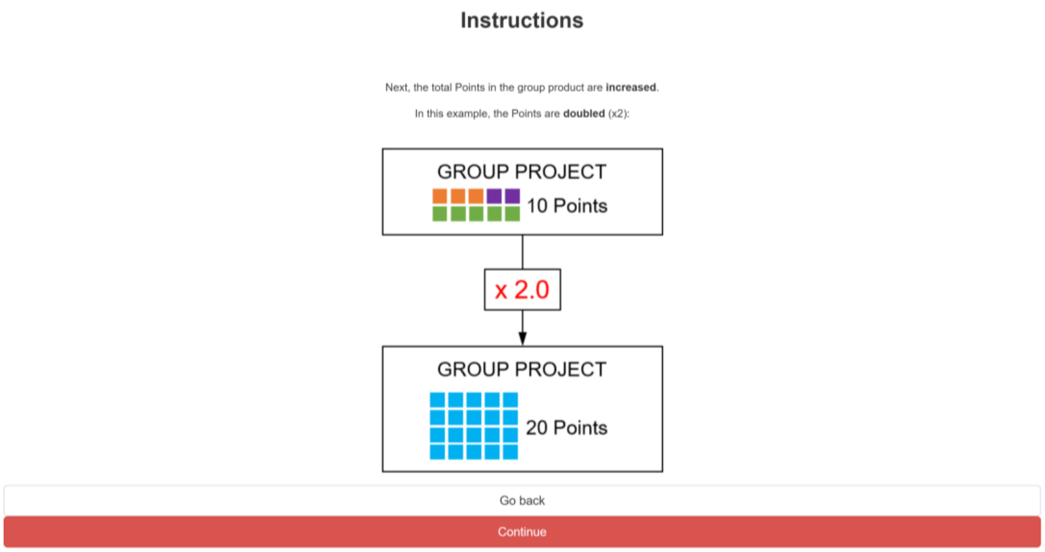

Fig S5. Instructions screen 4.

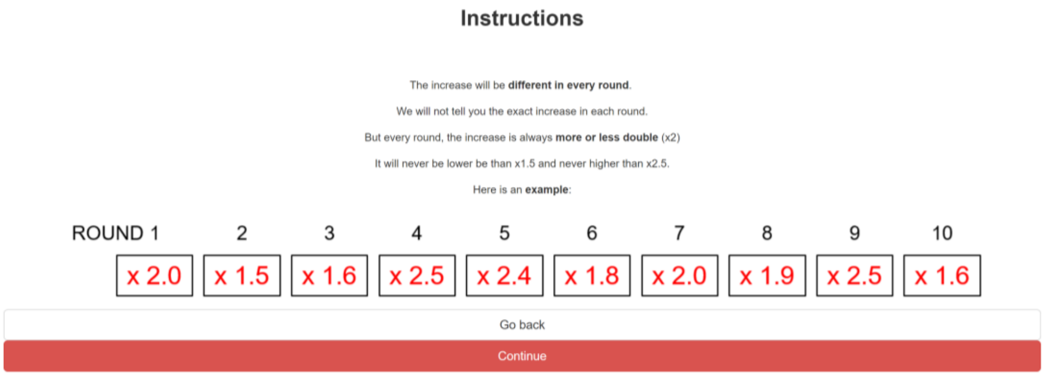

Fig S6. Instructions screen 5.

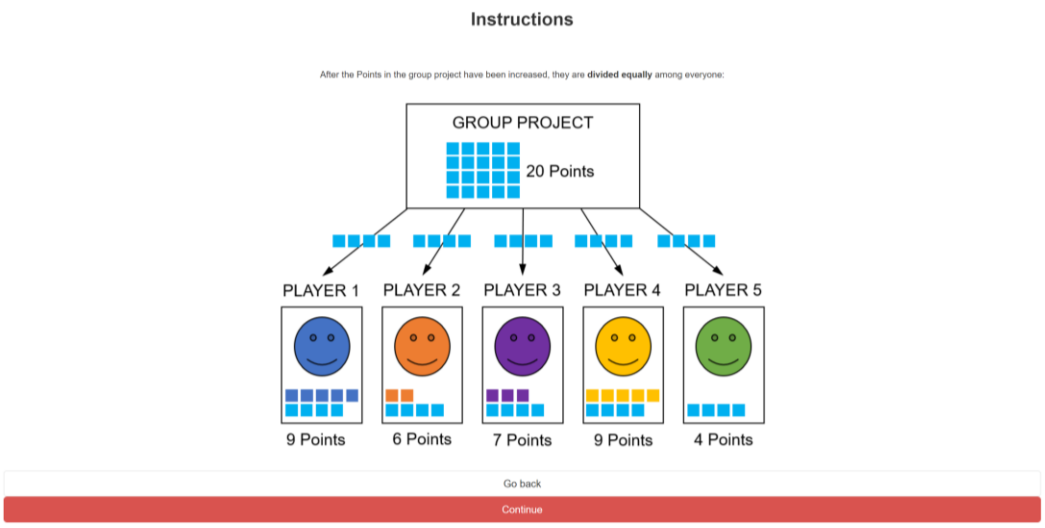

Fig S7. Instructions screen 6.

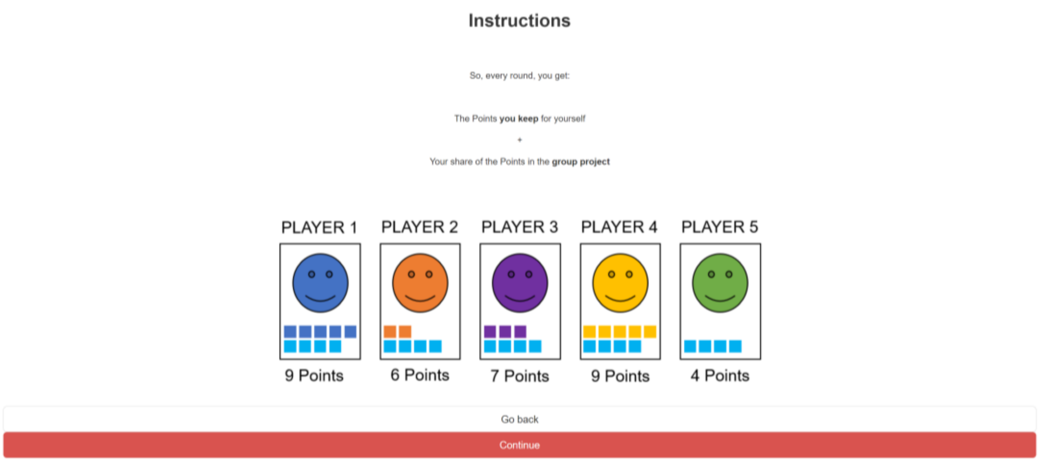

Fig S8. Instructions screen 7.

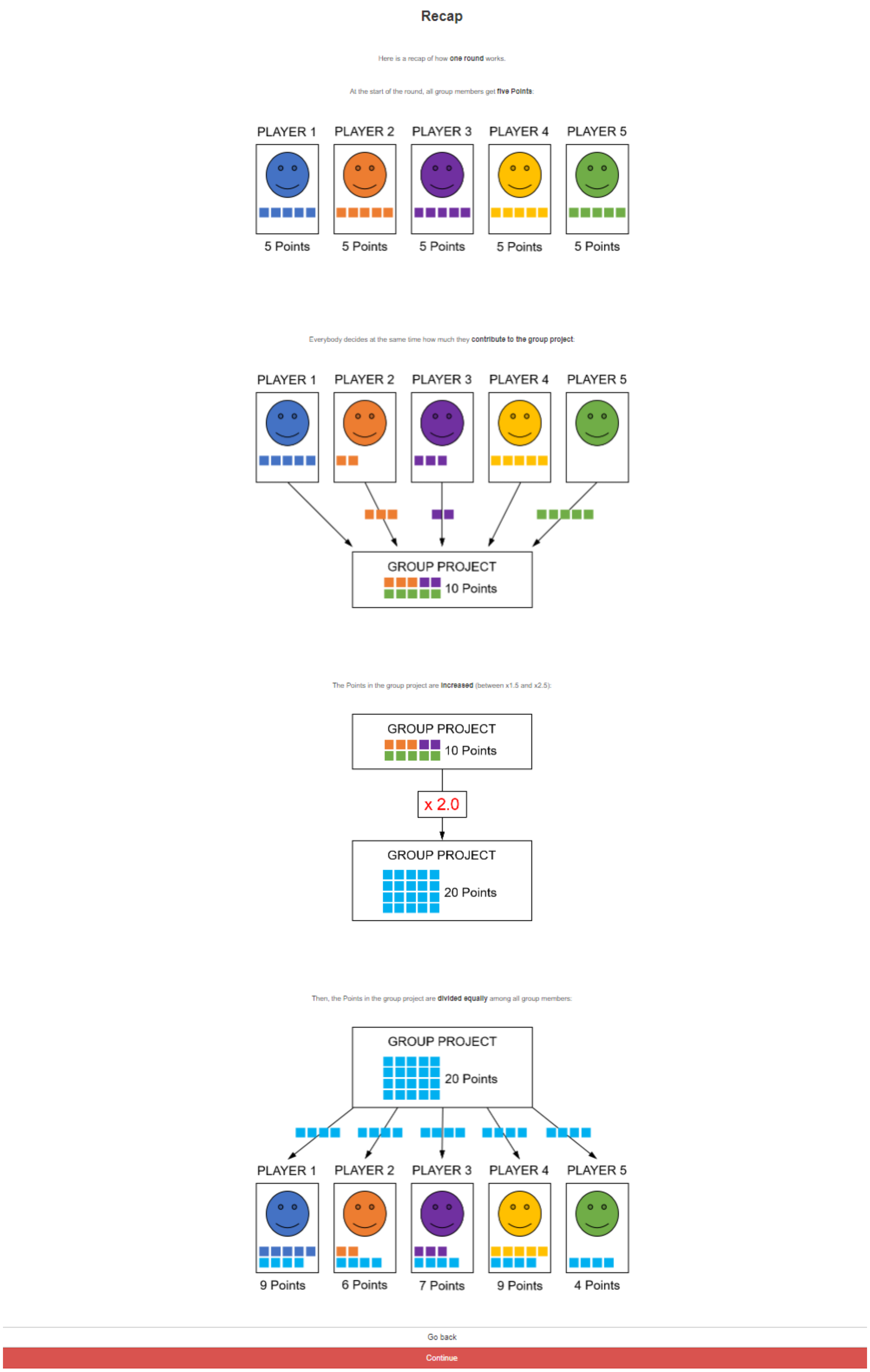

Fig S9. Recap screen.

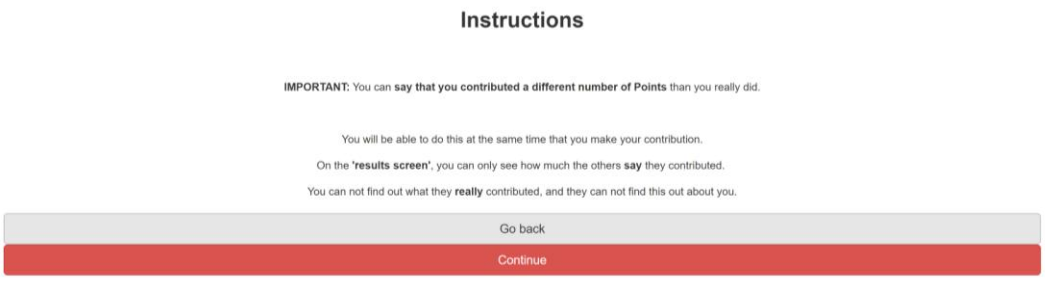

Fig S10. Instructions screen 8 (treatments with misrepresentation only).

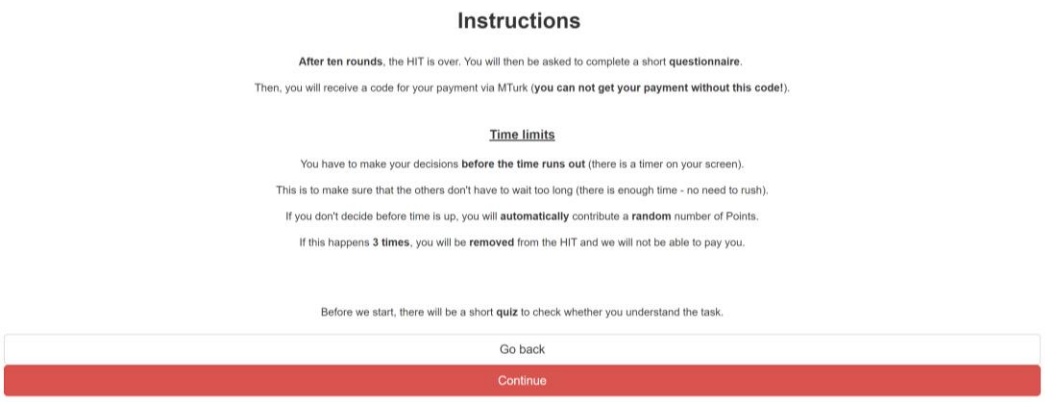

Fig S11. Instructions screen 9.

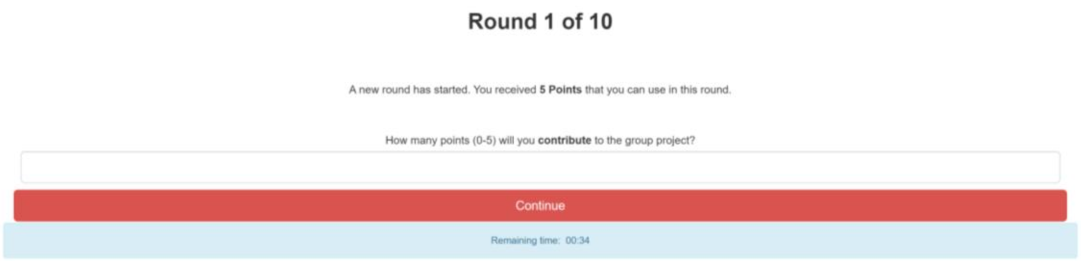

Fig S12. Decision screen for the treatments without misrepresentation.

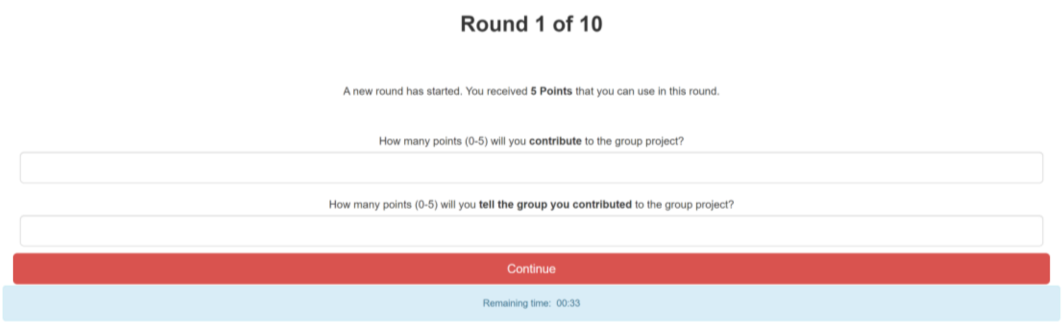

Fig S13. Decision screen for the treatments with misrepresentation.

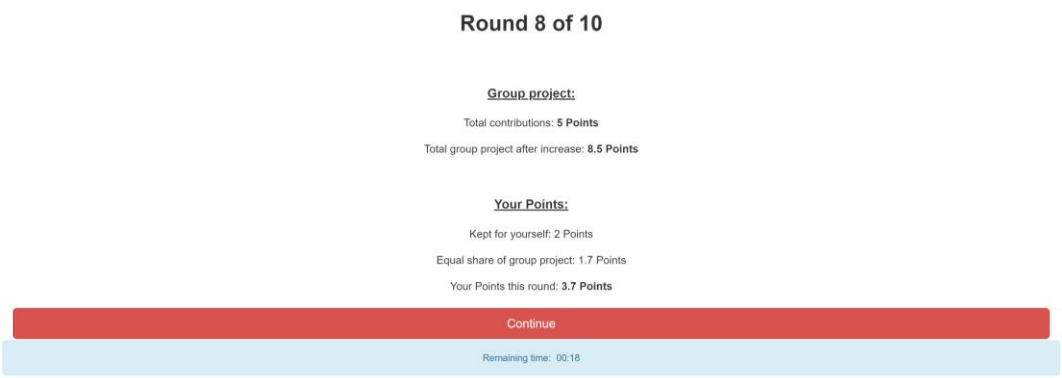

Fig S14. Results screen for the treatment without misrepresentation and without reputation.

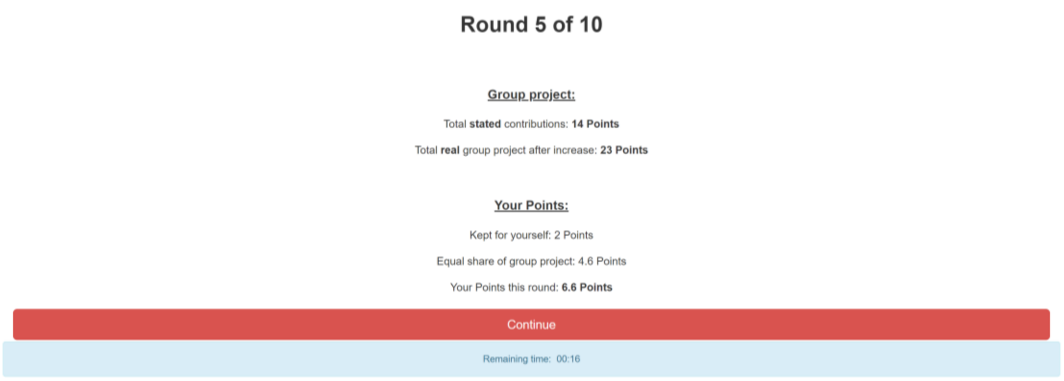

Fig S15. Results screen for the treatment with misrepresentation and without reputation.

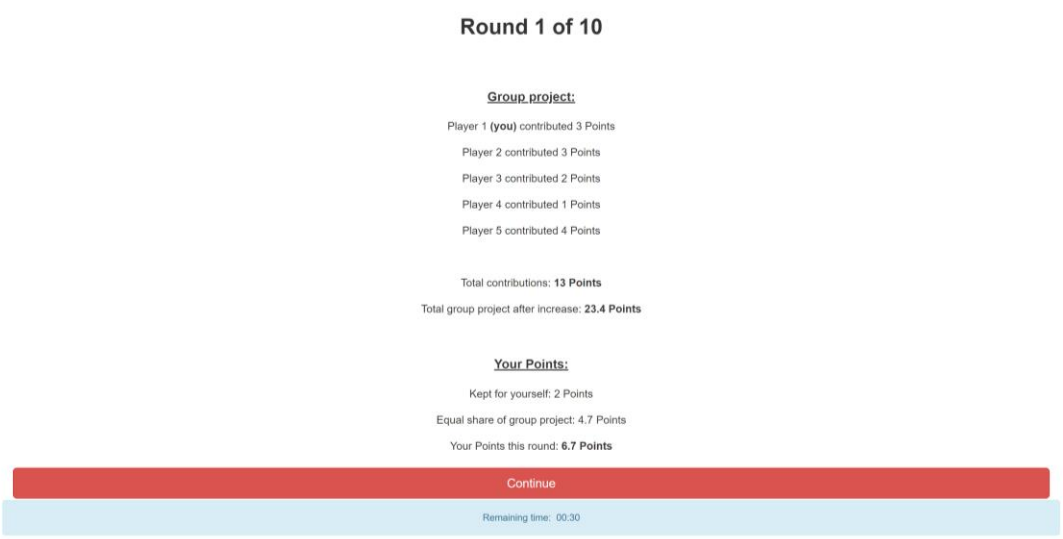

Fig S16. Results screen for the treatment without misrepresentation and with reputation.

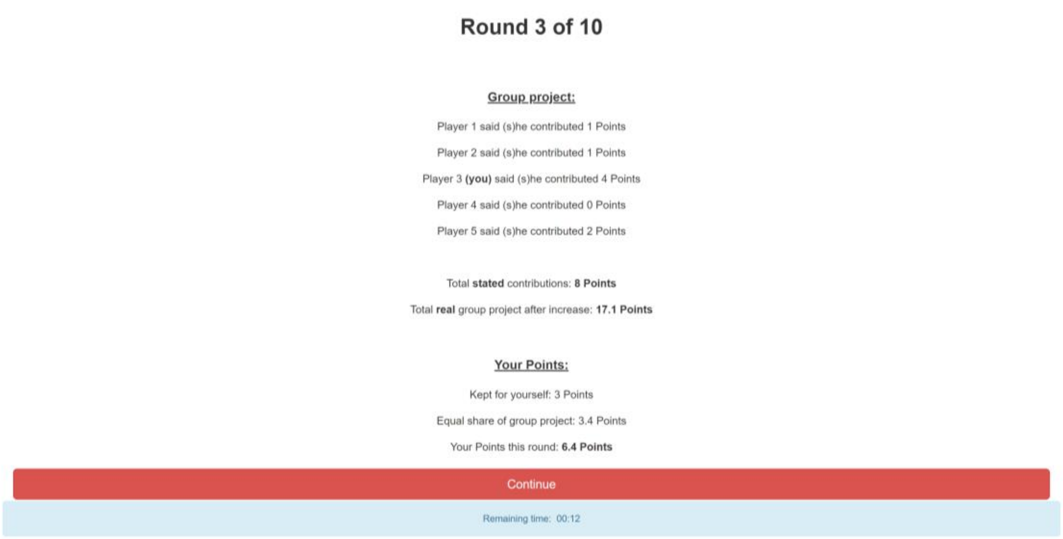

Fig S17. Results screen for the treatment with misrepresentation and with reputation.

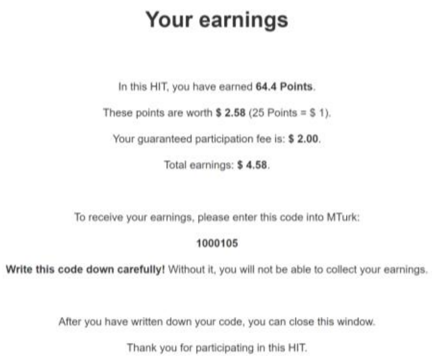

Your earnings

In this HIT, you have earned **64.4 Points**.

These points are worth **$ 2.58** (25 Points = $ 1).

Your guaranteed participation fee is: **$ 2.00**.

Total earnings: **$ 4.58**.

To receive your earnings, please enter this code into MTurk:

1000105

Write this code down carefully! Without it, you will not be able to collect your earnings.

After you have written down your code, you can close this window.

Thank you for participating in this HIT.

Fig S18. Final screen.

2. Comprehension questions to test participants

To make sure participants understood the instructions, there was a page with five multiple-choice comprehension questions after the participants finished all instruction pages. Once they had answered all questions, participants could 'submit' the form. If there were any mistakes, the participants would be explicitly shown which questions they answered incorrectly (without being shown what the right answers were), at which point they could change their answers and could submit again. Participants could only continue in the experiment once they had answered all comprehension questions correctly. If they submitted the form wrongly 10 times, they could not continue with the experiment. The questions (and possible responses) were formulated as follows (bold answers are correct):

Q1. After the Points in the group project have been increased, how are they divided among all group members?
A. Group members who contributed more to the group project receive more Points
B. All group members receive the same number of Points from the group project
C. The Points from the group project are randomly divided between all group members
D. Group members who contributed less to the group project receive more Points

Q2. After everybody has contributed, the Points in the group project are increased. By how much?
A. The Points are increased by x2.0 in every round
B. The Points are increased by a different number every round, and it can be any number
C. The Points are increased by a different number every round, but always between x1.5 and x2.5
D. The Points are increased by a number between x1.5 and x2.5, and it is the same number in every round

Q3. True or false? If you contribute more than 0 Points to the group project, you will have more than 5 Points at the end of the round.
A. True
B. False
C. It depends on how many Points the other group members contributed

Q4. In which of the following situations do you end up with the most points?
1. All five group members contributed 0 Points.
2. All five group members contributed 3 Points.
A. In situation 1
B. In situation 2
C. I would earn the same number of Points in both situations

Q5. In which of the following situations do you end up with the most points?
1. All other group members contributed 3 Points, but you contributed 0 Points.
2. All group members (including you) contributed 3 Points.
A. In situation 1
B. In situation 2
C. I would earn the same number of Points in both situations

3. Details of statistical models

Our main text presents the results of three statistical models, referred to in the Materials and Methods section as ‘Model 1’, ‘Model 2’ and ‘Model 3’. Figures S19-22 show effect plots of Model 1, visualizing the effects of the predictor variables on contributions. Tables S1 and S2 present the coefficients of this model as well as an Anova-table that specifies the significance of all effects. Figures S23-25 show effects plots of Model 2, and Tables S3 and S4 present its coefficients and Anova-table. Figures S26-28 show effects plots of Model 3, and Tables S5 and S6 present its coefficients and Anova-table.

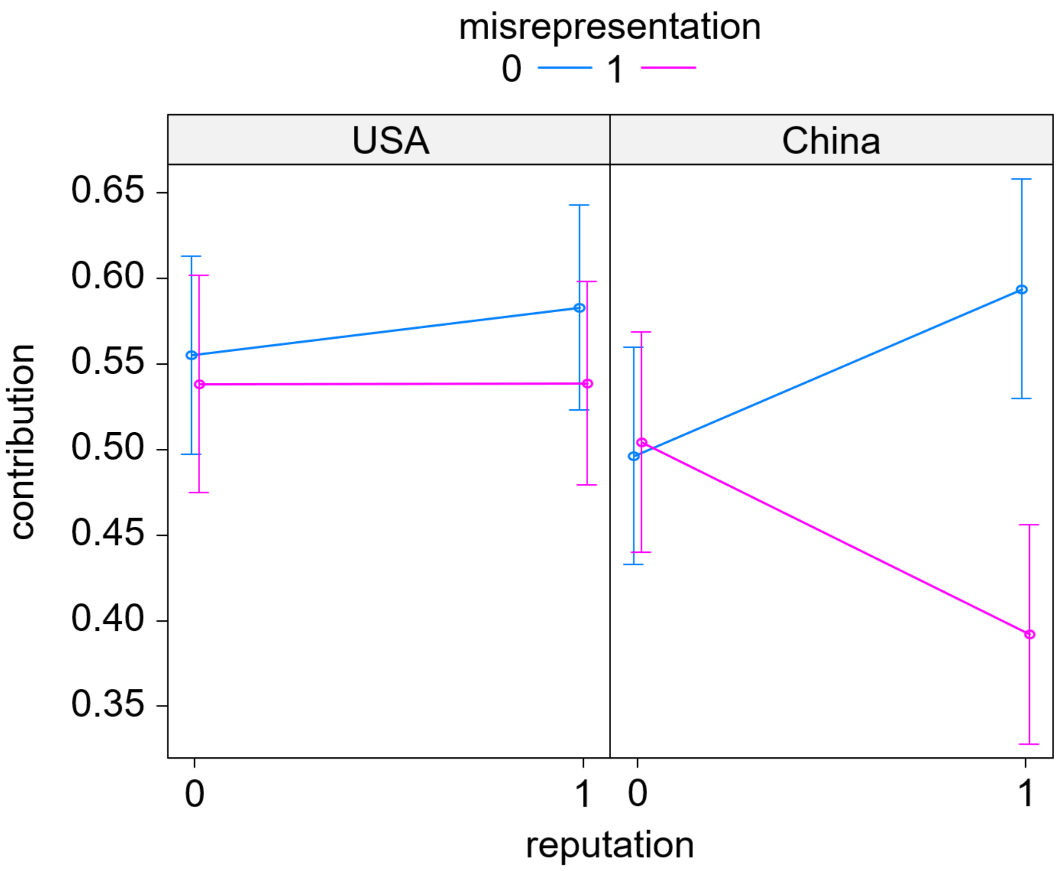

Fig S19. Effect plot of Model 1 for the variables ‘misrepresentation’, ‘reputation’, ‘culture’, and their interaction.

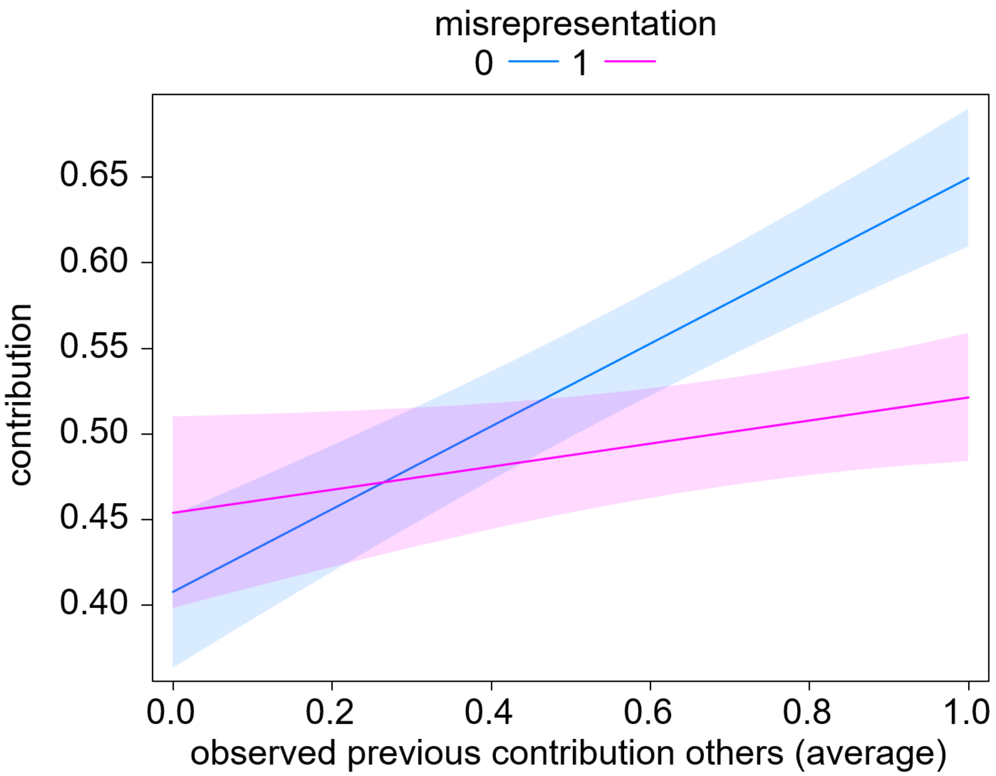

Fig S20. Effect plot of Model 1 for the variables 'misrepresentation', 'observed previous contribution others' and their interaction.

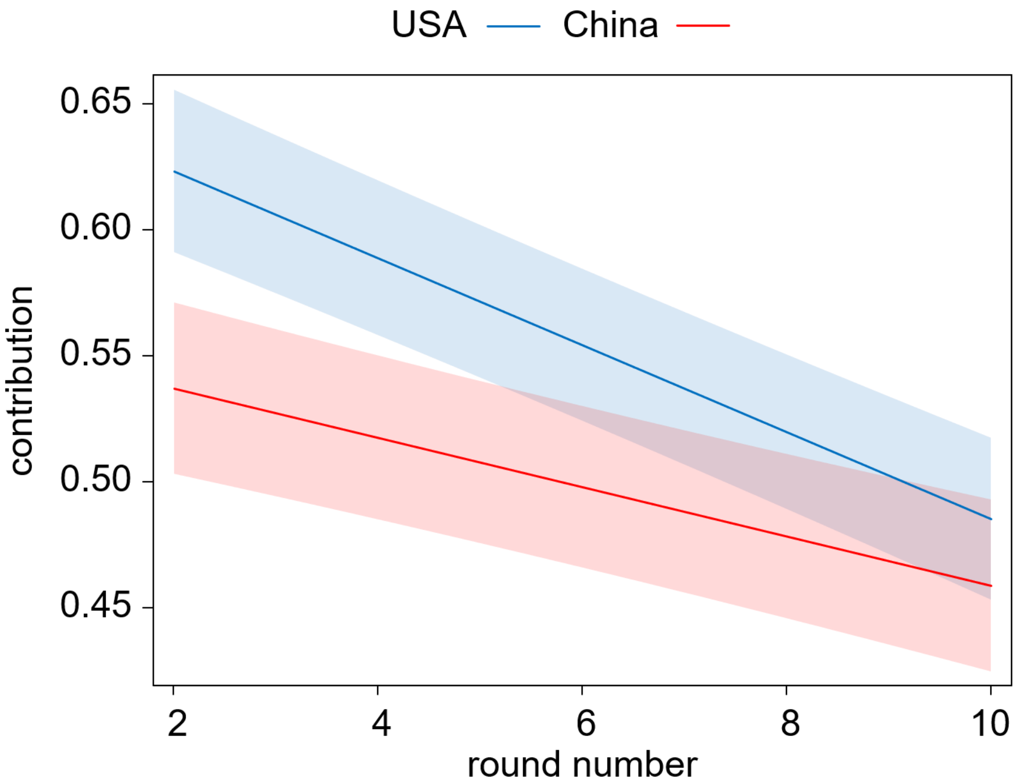

Fig S21. Effect plot of Model 1 for the variables ‘culture’, ‘round number’ and their interaction.

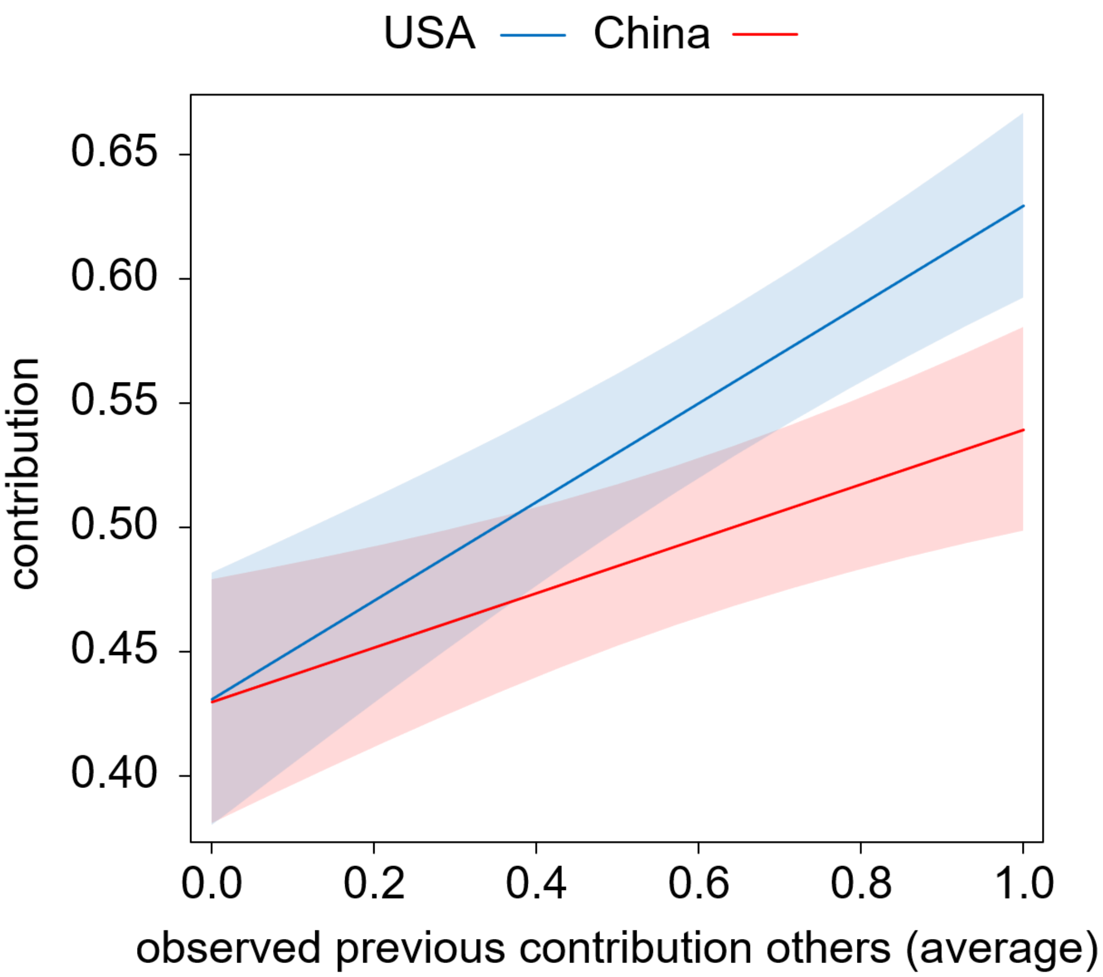

Fig S22. Effect plot of Model 1 for the variables ‘culture’, ‘observed previous contribution others’ and their interaction.

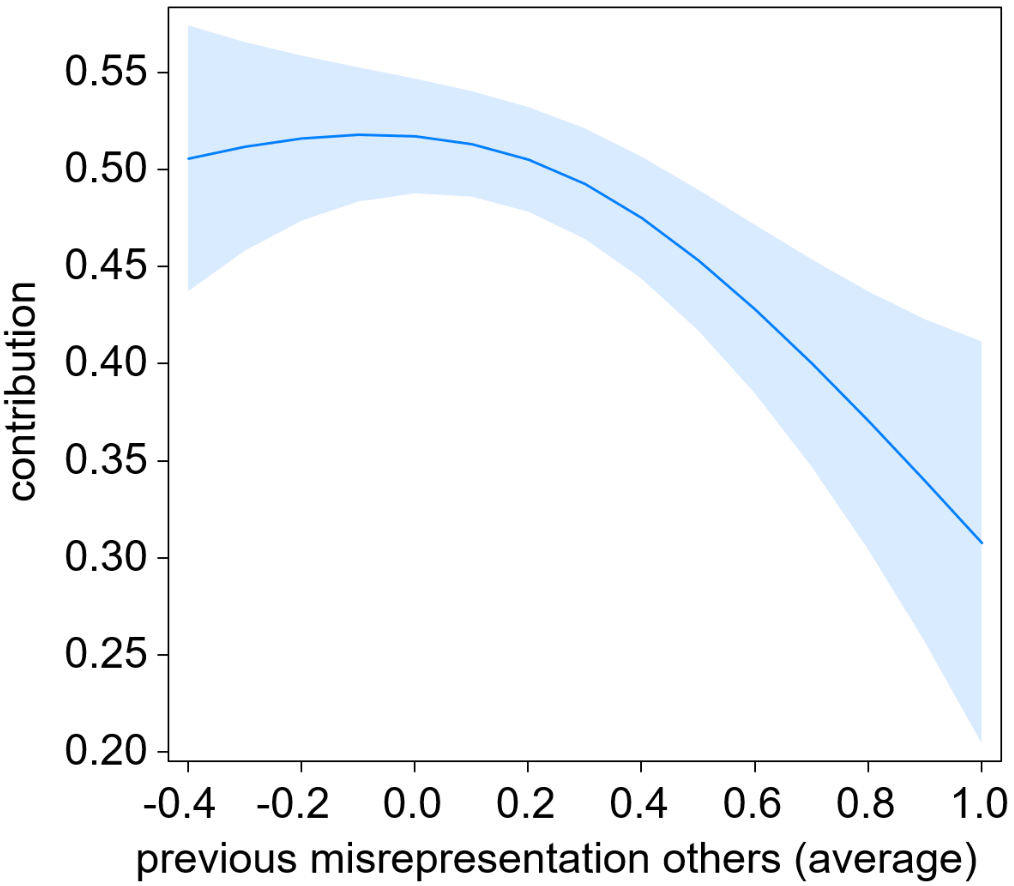

Fig S23. Effect plot of Model 2 for the variable ‘previous average misrepresentation others’.

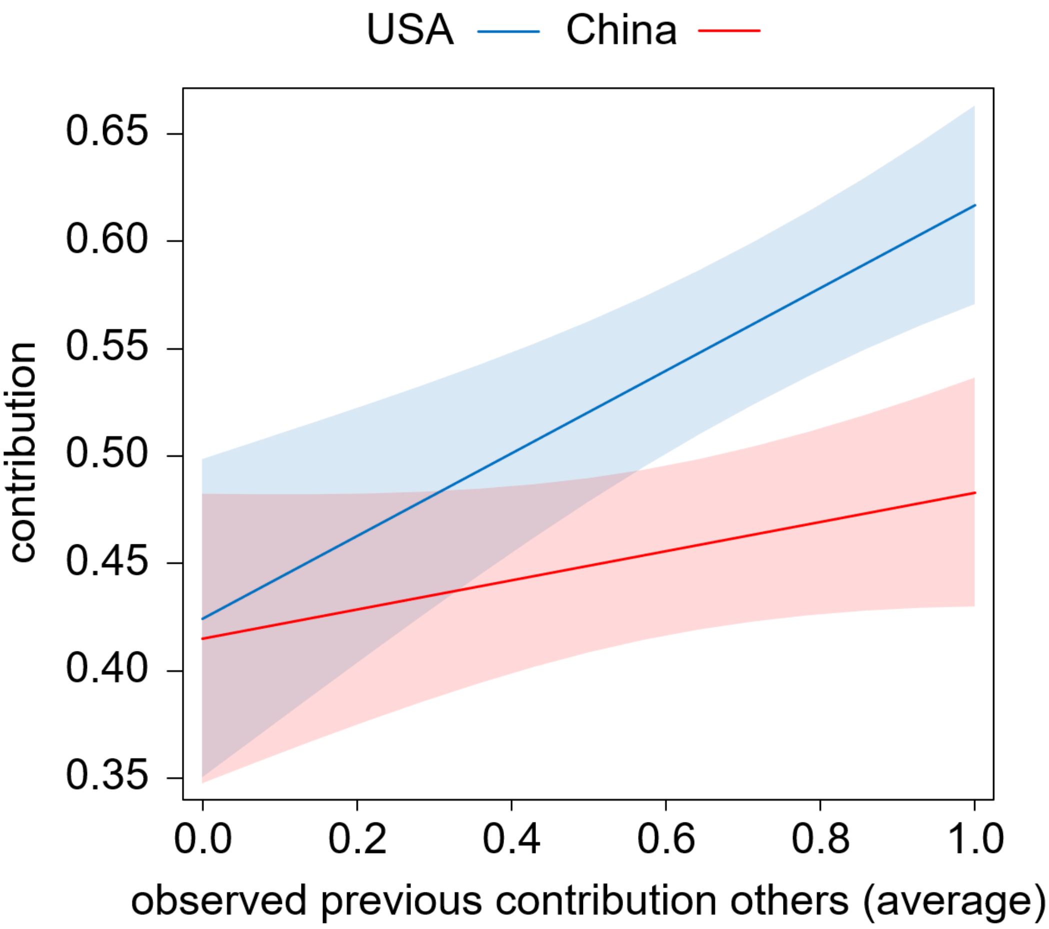

Fig S24. Effect plot of Model 2 for the variables 'culture' and 'observed previous contribution others'.

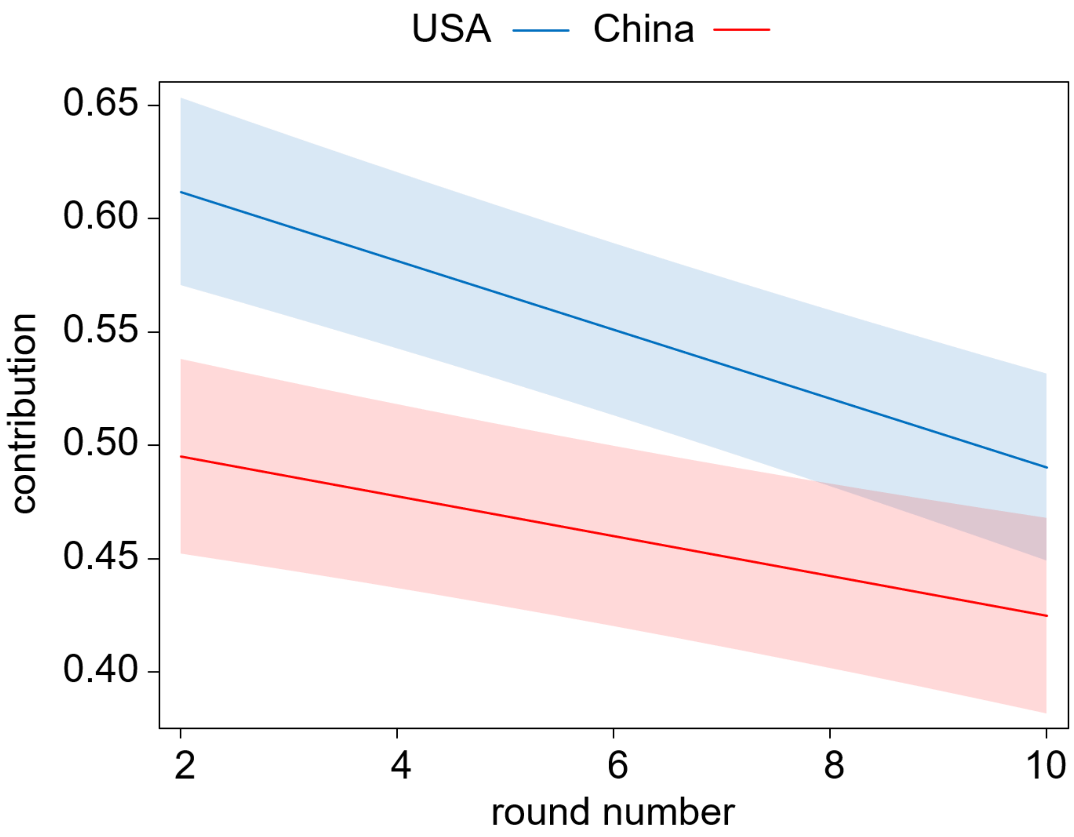

Fig S25. Effect plot of Model 2 for the variables ‘culture’ and ‘round number’.

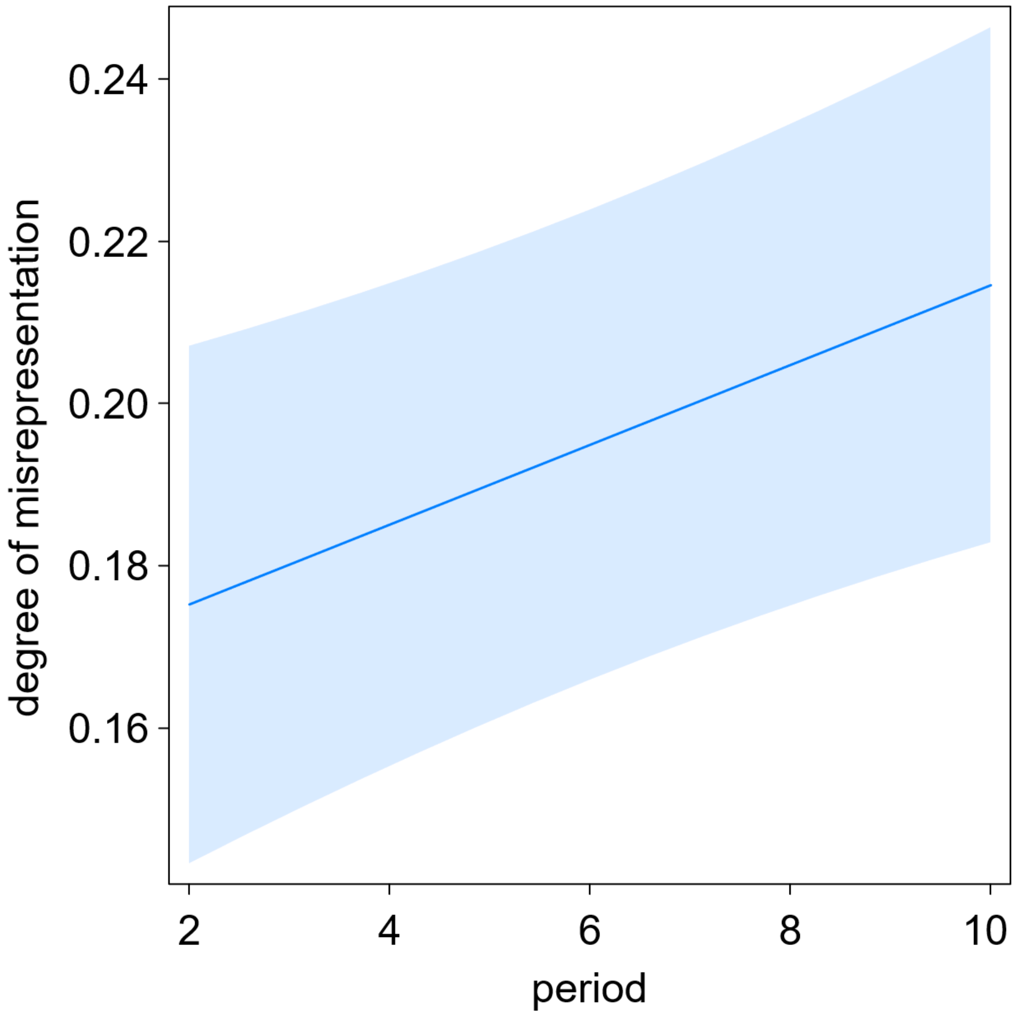

Fig S26. Effect plot of Model 3 for the variable ‘period’.

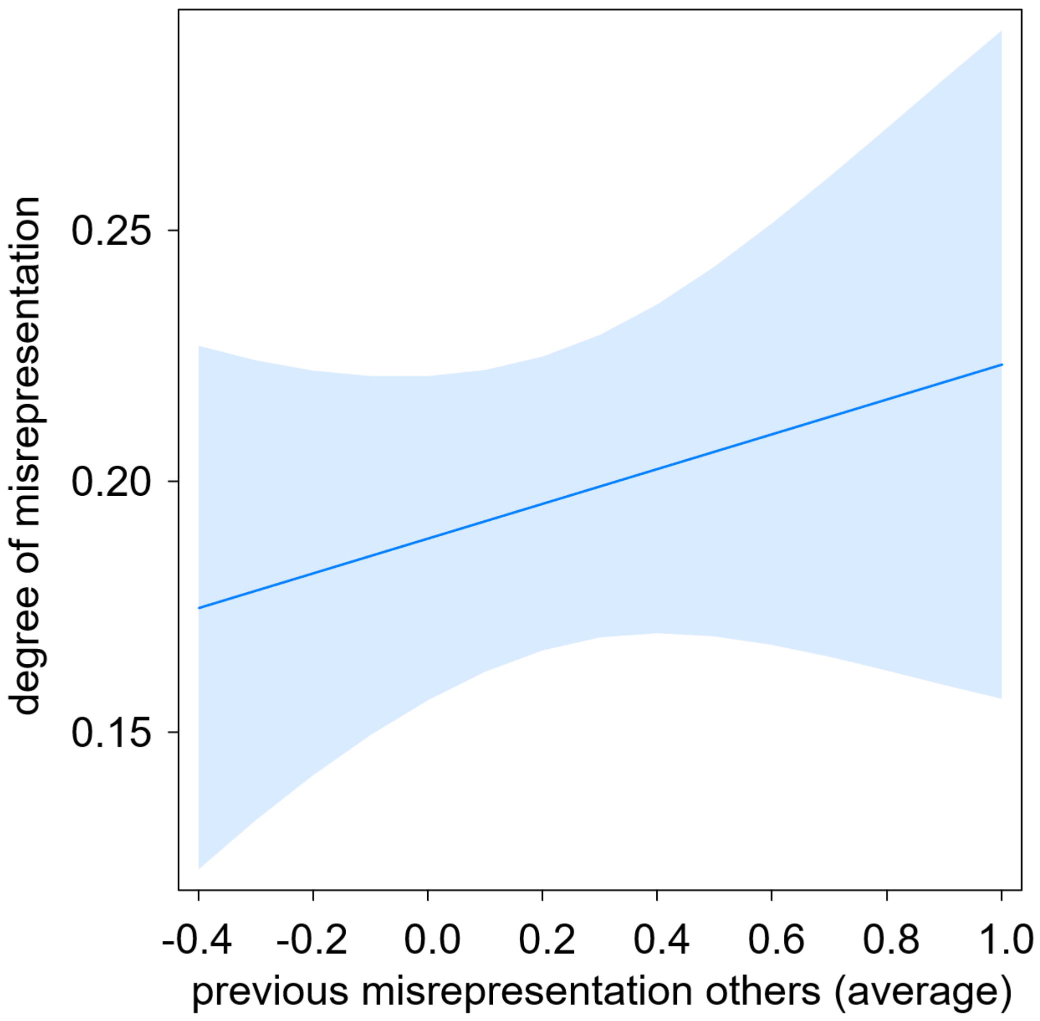

Fig S27. Effect plot of Model 3 for the variable ‘previous average misrepresentation others’.

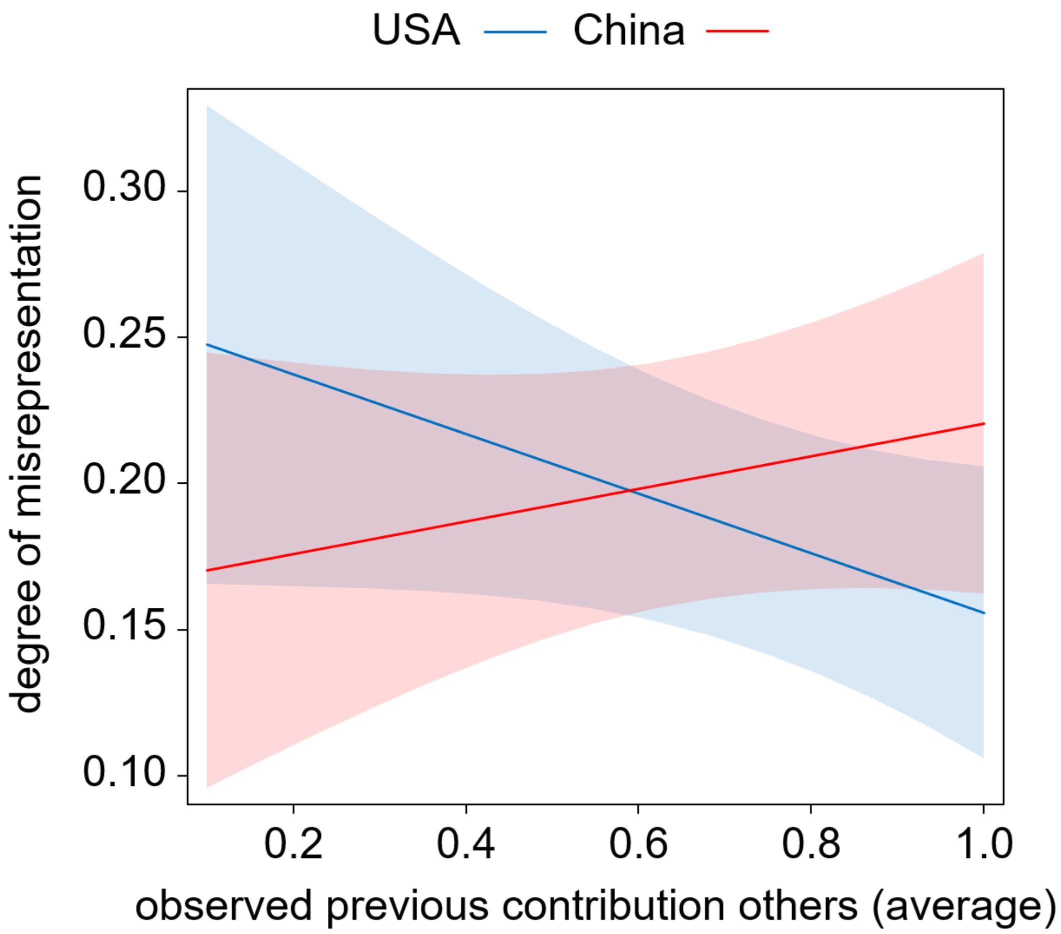

Fig S28. Effect plot of Model 3 for the variables ‘culture’ and ‘observed previous contribution others’.

```
Random effects:
 Groups     Name        Variance Std.Dev.
 ID:groupID (Intercept) 0.057775 0.24036
 groupID    (Intercept) 0.008726 0.09341
 Residual               0.052716 0.22960
Number of obs: 7734, groups:  ID:groupID, 870; groupID, 174

Fixed effects:
                     Estimate Std. Error        df t value Pr(>|t|)
(Intercept)         4.832e-01  3.741e-02  3.303e+02  12.918  < 2e-16 ***
round              -1.724e-02  1.431e-03  6.956e+03 -12.047  < 2e-16 ***
reput               2.776e-02  4.239e-02  1.444e+02   0.655 0.513615
misrep              9.102e-02  5.287e-02  2.951e+02   1.721 0.086228 .
culture            -4.848e-02  5.141e-02  2.661e+02  -0.943 0.346481
prevcon             2.835e-01  3.692e-02  4.430e+03   7.680 1.95e-14 ***
round:culture       7.455e-03  2.053e-03  6.904e+03   3.631 0.000284 ***
reput:misrep       -2.713e-02  6.097e-02  1.443e+02  -0.445 0.657003
reputat:culture     6.977e-02  6.246e-02  1.447e+02   1.117 0.265871
misrep:culture      2.491e-02  6.373e-02  1.494e+02   0.391 0.696395
misrep:prevcon     -1.744e-01  4.535e-02  6.049e+03  -3.845 0.000122 ***
culture:prevcon    -8.899e-02  4.522e-02  5.754e+03  -1.968 0.049115 *
reput:misrep:china -1.826e-01  8.936e-02  1.449e+02  -2.044 0.042797 *
---
Signif. codes:  0 ‘***’ 0.001 ‘**’ 0.01 ‘*’ 0.05 ‘.’ 0.1 ‘ ’ 1
```

Table S1. Coefficients of Model 1. Variable names: “round” indicates round number, “reput” indicates reputation treatment, “misrep” indicates misrepresentation treatment, “culture” indicates culture (China or USA) and “prevcon” indicates the observed average contribution of all interaction partners in the previous round.

```
                      Chisq Df Pr(>Chisq)    
(Intercept)          166.8704  1  < 2.2e-16 ***
round                145.1337  1  < 2.2e-16 ***
reput                  0.4288  1  0.5125732    
misrep                 2.9632  1  0.0851799 .  
culture                0.8895  1  0.3456252    
prevcon               58.9804  1  1.592e-14 ***
round:culture         13.1868  1  0.0002819 ***
reput:misrep           0.1980  1  0.6563367    
reput:culture          1.2476  1  0.2640195    
misrep:culture         0.1528  1  0.6958377    
misrep:prevcon        14.7814  1  0.0001207 ***
culture:prevcon        3.8730  1  0.0490673 *  
reput:misrep:culture   4.1766  1  0.0409857 *  
---
Signif. codes:  0 ‘***’ 0.001 ‘**’ 0.01 ‘*’ 0.05 ‘.’ 0.1 ‘ ’ 1
```

Table S2. The outcome of a Type III Anova on Model 1. This tests for the presence of an effect of each of the fixed predictors after the other predictors in the model. See the caption of Table S1 above for an explanation of the variable names.

```
Random effects:
 Groups     Name        Variance Std.Dev.
 ID:groupID (Intercept) 0.07333  0.2708
 Residual               0.05259  0.2293
Number of obs: 3765, groups:  ID:groupID, 425

Fixed effects:
                       Estimate Std. Error         df t value Pr(>|t|)
(Intercept)           4.930e-01  4.978e-02  3.406e+03   9.904  < 2e-16 ***
culture              -3.425e-02  5.739e-02  2.825e+03  -0.597   0.5507
prevcon               2.139e-01  5.131e-02  3.757e+03   4.170 3.12e-05 ***
round                -1.520e-02  2.065e-03  3.370e+03  -7.363 2.26e-13 ***
ns(prevmisrep,df=2)1 -8.960e-02  6.454e-02  3.737e+03  -1.388   0.1652
ns(prevmisrep,df=2)2 -2.126e-01  5.316e-02  3.738e+03  -3.999 6.48e-05 ***
culture:prevcon      -1.382e-01  6.777e-02  3.747e+03  -2.039   0.0415 *
culture:round         6.424e-03  2.919e-03  3.343e+03   2.201   0.0278 *
---
Signif. codes:  0 ‘***’ 0.001 ‘**’ 0.01 ‘*’ 0.05 ‘.’ 0.1 ‘ ’ 1
```

Table S3. Coefficients of Model 2. Note that the estimates and significance of the separate spline terms are given for completeness but not straightforward to interpret – the significance terms of the Anova table (Table S4) gives more direct information about the significance of the effects of the different variables. Variable names: “round” indicates round number, “reput” indicates reputation treatment, “culture” indicates culture (China or USA), “prevcon” indicates the observed average contribution of all interaction partners in the previous round and “prevmisrep” indicates the average misrepresentation of all interaction partners in the previous round.

```
                      Chisq Df Pr(>Chisq)
(Intercept)         98.0900  1  < 2.2e-16 ***
culture              0.3562  1  0.5506294
prevcon             17.3879  1  3.048e-05 ***
round               54.2083  1  1.803e-13 ***
ns(prevmisrep,df=2) 16.5420  2  0.0002558 ***
culture:prevcon      4.1568  1  0.0414672 *
culture:round        4.8429  1  0.0277597 *
---
Signif. codes:  0 ‘***’ 0.001 ‘**’ 0.01 ‘*’ 0.05 ‘.’ 0.1 ‘ ’ 1
```

Table S4. The outcome of a Type III Anova on Model 2. This tests for the presence of an effect of each of the fixed predictors after the other predictors in the model. See the caption of Table S3 above for an explanation of the variable names.

```
Random effects:
 Groups     Name        Variance  Std.Dev.
 ID:groupID (Intercept) 8.359e-02 0.28913
 groupID    (Intercept) 9.429e-05 0.00971
 Residual               6.765e-02 0.26010
Number of obs: 3764, groups:  ID:groupID, 425; groupID, 85

Fixed effects:
                  Estimate Std. Error         df t value Pr(>|t|)
(Intercept)      2.216e-01  4.714e-02  8.139e+02   4.701 3.04e-06 ***
culture         -9.288e-02  6.035e-02  6.426e+02  -1.539  0.12432
round            4.928e-03  1.676e-03  3.366e+03   2.940  0.00331 **
prevmisrep       3.457e-02  3.763e-02  2.448e+03   0.919  0.35836
prevcon         -1.017e-01  5.771e-02  2.453e+03  -1.762  0.07820 .
culture:prevcon  1.575e-01  7.645e-02  2.796e+03   2.060  0.03945 *
---
Signif. codes:  0 ‘***’ 0.001 ‘**’ 0.01 ‘*’ 0.05 ‘.’ 0.1 ‘ ’ 1
```

Table S5. Coefficients of Model 3. Variable names: “round” indicates round number, “culture” indicates culture (China or USA), “prevcon” indicates the observed average contribution of all interaction partners in the previous round and “prevmisrep” indicates the average misrepresentation of all interaction partners in the previous round.

```
                 Chisq Df Pr(>Chisq)    
(Intercept)     22.0975  1  2.591e-06 ***
culture          2.3683  1   0.123823    
round            8.6432  1   0.003283 ** 
prevmisrep       0.8440  1   0.358266    
prevcon          3.1045  1   0.078077 .  
culture:prevcon  4.2452  1   0.039361 *  
---
Signif. codes:  0 ‘***’ 0.001 ‘**’ 0.01 ‘*’ 0.05 ‘.’ 0.1 ‘ ’ 1
```

Table S6. The outcome of a Type III Anova on Model 3. This tests for the presence of an effect of each of the fixed predictors after the other predictors in the model. See the caption of Table S5 above for an explanation of the variable names.

4. Graphs of contributions over time

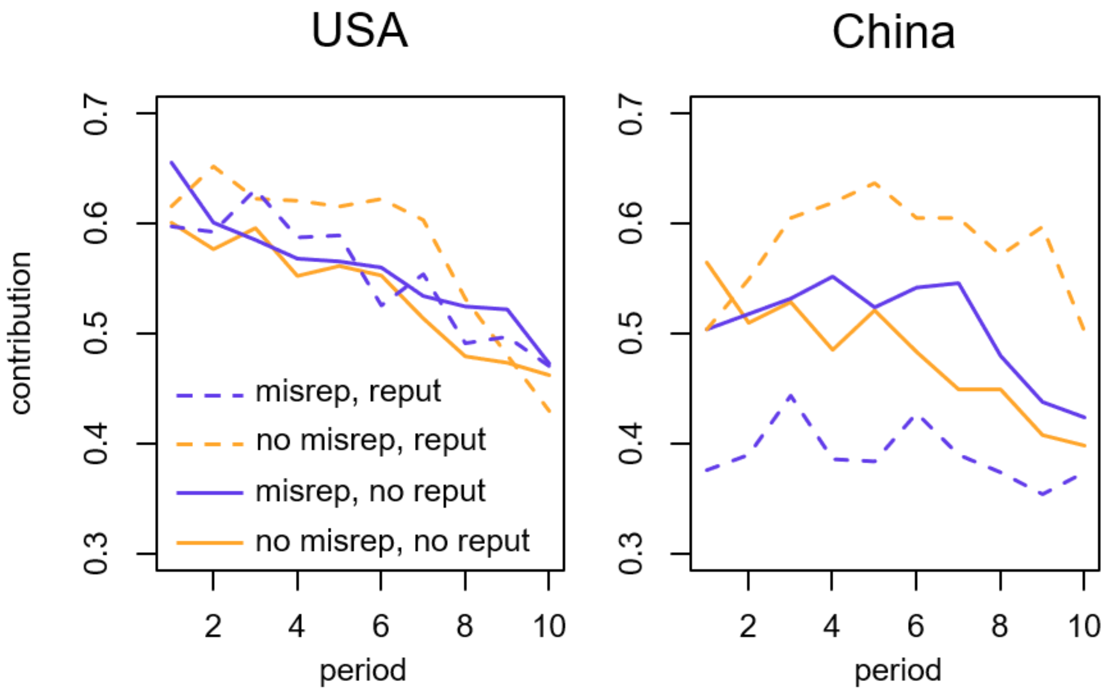

Figure S29. Contributions over time. Lines represent the average individual contributions for each treatment (indicated by different colours and line types) over the 10 periods of the experiment.
